# Implicit and explicit social mentalizing: dual processes driven by a shared neural network

**DOI:** 10.3389/fnhum.2013.00560

**Published:** 2013-09-13

**Authors:** Frank Van Overwalle, Marie Vandekerckhove

**Affiliations:** Department of Psychology, Vrije Universiteit BrusselBrussels, Belgium

**Keywords:** mentalizing, implicit inferences, fMRI, ERP, dual-process theories

## Abstract

Recent social neuroscientific evidence indicates that implicit and explicit inferences on the mind of another person (i.e., intentions, attributions or traits), are subserved by a shared mentalizing network. Under both implicit and explicit instructions, ERP studies reveal that early inferences occur at about the same time, and fMRI studies demonstrate an overlap in core mentalizing areas, including the temporo-parietal junction (TPJ) and the medial prefrontal cortex (mPFC). These results suggest a rapid shared implicit intuition followed by a slower explicit verification processes (as revealed by additional brain activation during explicit vs. implicit inferences). These data provide support for a default-adjustment dual-process framework of social mentalizing.

## Introduction

Tell me what you did today, and I'll tell what you want and who you are. Behaviors are quite often the main road to enter people's mind, to infer their intentions and judge their personality traits. Social inferences that rely on insights about other people's mental content such as intentions, desires, beliefs, traits or other high-level characteristics are termed *mentalizing* inferences. Contrary to the old idea that such complex inferences need a lot of explicit mental elaboration, behavioral research in the 80s (Winter and Uleman, 1984) documented that such person inferences, including trait inferences, are often made implicitly and automatically, without awareness or control about the inference process. The fascination for such implicit and rapid inferences comes from the fact they are relatively correct based on brief verbal or non-verbal information (Letzring et al., 2006), although they may at times differ from explicit inferences. What is the true inference then? The aim of the present paper is to demonstrate that current neuroscientific evidence on mentalizing suggests that implicit and explicit person inferences do not rely on strictly distinct neural processes or substrates.

To explain the distinction between implicit and explicit social judgments, social cognition researchers drew parallels with similar distinctions in cognitive psychology, collectively termed dual-process theories (for reviews see Evans, 2008; Evans and Stanovich, 2013). Dual-process theories argue that there are two different modes of processing, termed *implicit* (also termed unconscious, automatic, spontaneous, experiential, heuristic, intuitive, impulsive, and reflexive) and *explicit* (also termed conscious, controlled, rational, systematic, analytical, and reflective; Schneider and Shiffrin, 1977; Chaicken, 1980; Epstein, 1994; Strack and Deutsch, 2004; Lieberman, 2007). The key feature of dual-process theories is that implicit processes are inaccessible to consciousness and control, while explicit processes are accessible to awareness, introspection and flexible control. It is also assumed that implicit processes are rapid, while explicit processes are slow (Evans, 2008). Some dual-process theorists in social cognition (and other domains) assume that implicit and explicit processes are subserved by different information processing systems (e.g., associative vs. rule-based; Smith and DeCoster, 2000) or subserved by different brain areas (e.g., Satpute and Lieberman, 2006; Lieberman, 2007; Forbes and Grafman, 2013).

## Do dual processes imply distinct networks?

Behavioral research in social cognition accumulated a growing body of evidence in favor of dual-process models by demonstrating that many person inferences occur not always explicitly, but often implicitly (for a review, Uleman, 1999). Recent findings demonstrate that representing other agents' beliefs, is an implicit capacity acquired early at 7 month of age (Kovacs et al., 2010) and implicitly sustained during adulthood (Schneider et al., 2012a), although it requires some minimal executive resources (Qureshi et al., 2010; Schneider et al., 2012b). However, the idea that implicit and explicit social mentalizing are subserved by distinct and exclusive processing systems or brain networks appears unjustified. True, some neural networks are mainly involved in implicit social processing. Recent neuroscientific research has uncovered subcortical mechanisms located in the amygdala and other limbic structures that elicit primitive affective reactions (e.g., rapid impressions of a face; Todorov et al., 2008a,b; Vandekerckhove and Panksepp, 2011; Forbes et al., 2012), as well as mirror-like neural networks responsible for the understanding of non-verbal movements and actions of humans (Iacoboni, 2009; Van Overwalle and Baetens, 2009).

**Figure 1:**
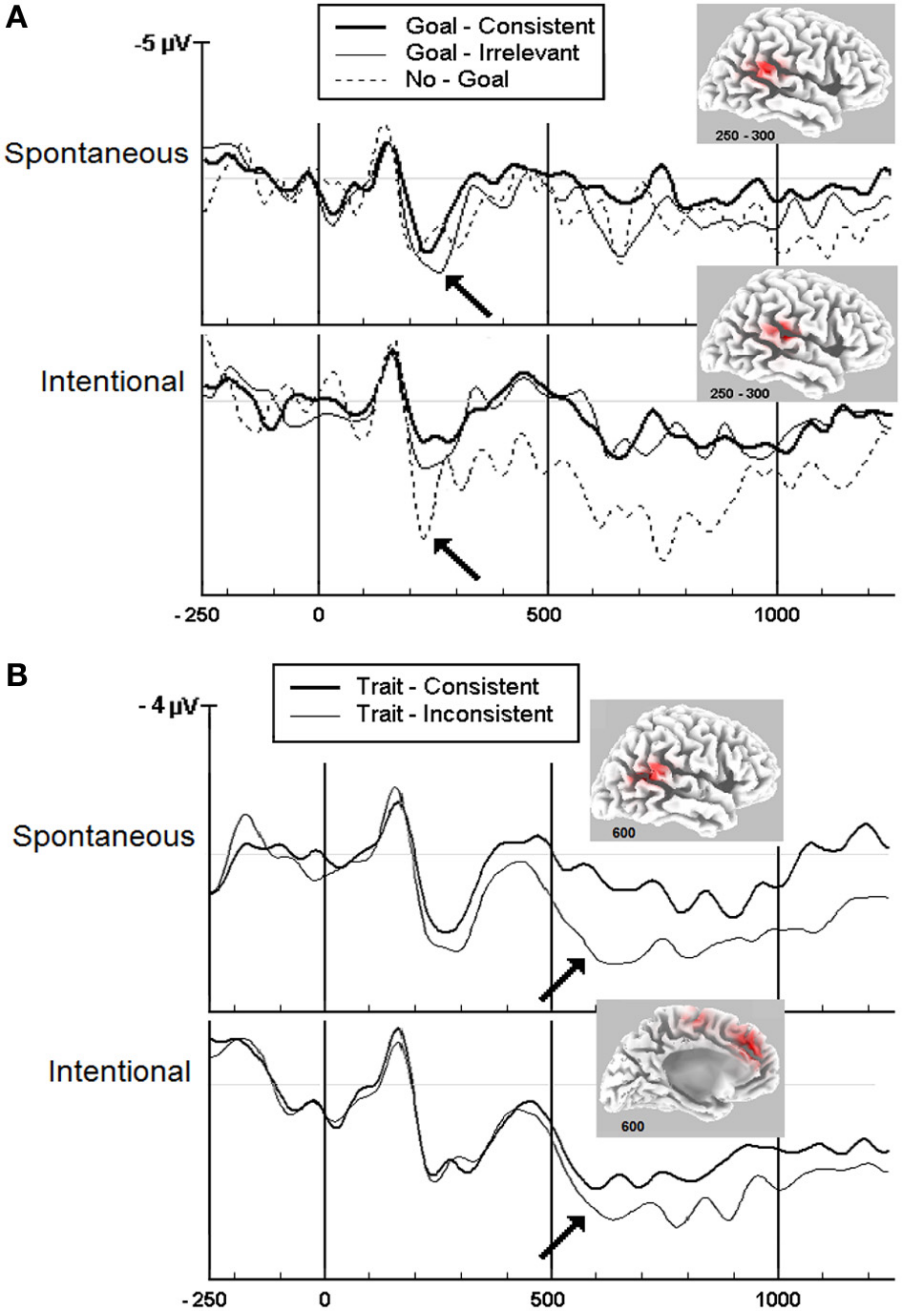
**Grand-averaged ERP waveforms showing positive deflections at the central midline (Cz) scalp sites given spontaneous and intentional instructions.** The timeline is given in ms. A positive amplitude is shown downward. **(A)** Goal inferences: Goal-consistent words diverge significantly from goal-irrelevant and no-goal words at about 250 ms (*p* < 0.05) as indicated by the arrows. Insets: LORETA source analysis of the TPJ at 250–300 ms. Adapted with permission from Van der Cruyssen et al. (2009). Copyright 2008 by Psychology Press. **(B)** Trait inferences: Trait-consistent words diverge significantly from trait-inconsistent words at about 600 ms (*p* < 0.05) as indicated by the arrows. Insets: LORETA source analysis of the TPJ and mPFC at 600 ms. Adapted with permission from Van Duynslaeger et al. (2007). Copyright 2007 by Oxford Press.

In contrast, it is acknowledged that higher-level mentalizing brain areas subserve computations that are neither exclusively implicit or explicit (Satpute and Lieberman, 2006; Keysers and Gazzola, 2007; Forbes and Grafman, 2013). On the contrary, the content of the social inference process seems more crucial than the nature of the process. Each core area in the mentalizing network seems responsible for a distinct computation and appears sensitive to a specific input. The *temporo-parietal junction* (TPJ) seems responsible for judgments on temporary beliefs and intentions, while the *medial prefrontal cortex* (mPFC) seems involved in enduring trait inferences and other stable characteristics (Figure 1; see for reviews, Amodio and Frith, 2006; Van Overwalle, 2009; Lombardo et al., 2011; Bzdok et al., 2012; Denny et al., 2012). Each of these areas supports computations of specific social judgments, but none seems predominantly recruited for explicit or implicit inferences.

## Implicit and explicit mentalizing share early timing and core brain areas

Methods that were commonly used to explore implicit processes in behavioral research, cannot always be directly adapted for neuroimaging research. For instance, subliminal presentation of stories or picture sequences to depict events is impossible, while the use of cognitive load may trigger unwanted neural processes that may interfere with the social inference task (e.g., Qureshi et al., 2010). To illustrate, in contrast to passively watching or explicitly empathizing with another person while reading social information, Morelli and Lieberman (2013) found reduced activity in the TPJ and mPFC when participants were instructed to memorize simultaneously an irrelevant number. Moreover, cognitive load by memorizing further increased activity in a host of areas related to episodic memory and executive control (i.e., precuneus, dorsal anterior and middle cingulate, lateral PFC and inferior parietal cortex). Critically, these increased activations occurred in comparison to either explicit or implicit mentalizing instructions, demonstrating that unlike behavioral research, cognitive load by a secondary task may actually complicate the understanding of neural processes rather than clarifying it. However, the mere presentation of behavioral descriptions that invite most participants to spontaneously infer a social goal, belief or trait under implicit reading instructions, as opposed to explicitly instructions to make these inferences, avoids these methodological difficulties. Nevertheless, passively reading (as well as secondary tasks) cannot eliminate entirely the possibility that some amount of explicit mentalizing might still occur. Using this approach, recent neuroimaging research converges on the view that there is a common underlying mentalizing network that is relatively blind to the implicit or explicit nature of the inference, and that seems more sensitive to the content of the inference.

### ERP timing: an identical onset for implicit and explicit inferences

Van Overwalle and colleagues documented that the neural timing (measured by event-related potentials or ERPs) of an early social inference is almost identical under implicit or explicit processing. Under implicit instructions, the participants were told that they had to “read carefully” the material, while participants under explicit instructions had to answer repeatedly the question: “what is the [inference] of this person?” Participants were divided in two separate groups, to avoid contamination of the explicit instructions on implicit judgments. Participants read a number of sentences that all implied the same inference (e.g., “nice” as a trait), and thus provided a strong impression on the target person. At the end of the trial, a critical sentence was presented that was either consistent with the earlier inference (e.g., “gave her sister a *hug*”), inconsistent (e.g., “gave her brother a *slap*”), or neutral (e.g., “gave her mother a *bottle*”). Because timing is crucial in ERP research, the sentences were presented word-by-word in the middle of the screen, and ERP timing started at the beginning of the critical word (see italics in the examples above) that diverged between conditions.

A first ERP study on goal inferences (Van der Cruyssen et al., 2009) reported that goal inferences were made after about 250 ms, again irrespective of the implicit or explicit instruction (see the significant divergence of wave patterns under consistent vs. inconsistent conditions, Figure 1A). A second ERP study on trait inferences (Van Duynslaeger et al., 2007) documented that the onset of trait inferences occurs at about 600 ms irrespective of instructions (Figure 1B). Source localization of the ERP waves suggested that the core mentalizing areas (TPJ and mPFC) were most strongly recruited (Figure 1 insets, using LORETA, Pascual-Marqui et al., 1994; Pascual-Marqui, 1999). Together, these result seems to provide support for a core single-system account with an identical onset at the beginning of mentalizing. Interestingly, the results also reveal that goal inferences are faster than trait inferences, consistent with the proposition by Van Overwalle (2009) that goals involve a quick evaluation of the here-and-now by the TPJ, while traits reflect slower abstractions by the mPFC extracted from behaviors identified in the TPJ (see also Van Overwalle et al., 2011; Ma et al., 2012b).

### fMRI localization: overlap between implicit and explicit inferences

Van Overwalle and colleagues conducted a series of functional imaging studies to explore the overlap between explicit and implicit mentalizing, using a very similar procedure as described before. The results showed significant overlap in mentalizing activity between instructions, even though brains from different participants were compared.

**Figure 2:**
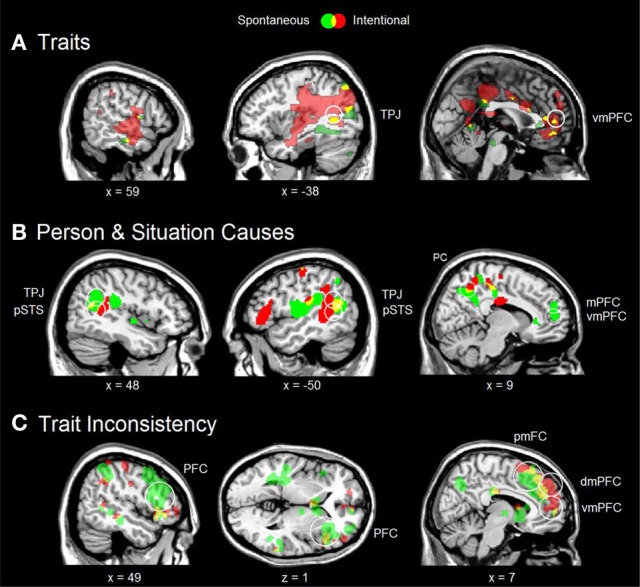
**Various social inferences under spontaneous (green) and intentional (red) instructions, and their overlap (yellow). (A)** Consistent trait > irrelevant trait contrast from Ma et al. (2011) with significant conjunction in the left TPJ (MNI coordinates −58 −58 32). **(B)** Person Cause > Baseline and Situation Cause > Baseline conjunction from Kestemont et al. (2013) with significant conjunction in the bilateral TPJ and pSTS (respective MNI coordinates 46 −56 20; −50 −54 18; 52 −56 14; −50 −56 16). **(C)** Inconsistent trait > Consistent trait contrast from Ma et al. (2012a) with significant conjunction in the mPFC (MNI coordinates 4 42 32). In all analyses, whole-brain activation was thresholded at *p* < 0.005 (uncorrected) with at least 10 voxels. Circles indicate regions of interest with significant activation after FDR correction at *p* < 0.10. vmPFC, ventral part of the mPFC; dmPFC, dorsal part of the mPFC; pmFC, posterior frontal cortex; PFC, lateral prefrontal cortex.

A first fMRI study on implicit and explicit trait inferences (Ma et al., 2011) revealed common activation in the left TPJ, mPFC and bilateral temporal pole (Figure 2A). This overlap was statistically significant for the TPJ, indicating a reliable common process across brains from different individuals. Importantly, there were also differences between instructions. Implicit trait inferences significantly recruited only the core mentalizing areas of the TPJ and mPFC, whereas explicit trait inferences additionally recruited other brain areas involved in mentalizing, including the precuneus (autobiographic memory) and posterior part of the superior temporal sulcus (pSTS; biological motion). Analogous findings were reported by Rameson et al. (2010) for implicit and explicit self-descriptions.

In a second fMRI study on person and situation causes (Kestemont et al., 2013), there was an overlap between implicit and explicit instructions in the bilateral TPJ and pSTS, and the precuneus, which was significant for the TPJ and pSTS (Figure 2B). Again, there were also differences. Only implicit inferences increased the activation of the mPFC, suggesting a tendency to make dispositional trait attributions to the person irrespective of the situational circumstances that may have constrained or induced the behavior of the agent, known as the *fundamental attribution bias* (Moran et al., unpublished). This biased activation of the mPFC was absent under explicit instructions, consistent with decreased biased processing documented in behavioral research (Gilbert and Malone, 1995) and neuroimaging (Brosch et al., 2013).

In a third fMRI study on trait inconsistencies (Ma et al., 2012a), a significant overlap was found in the dorsal part of the mPFC (Figure 2C). Like in the previous trait study, some brain areas were more active only under explicit instructions, including the left TPJ and pSTS (biological motion) and the precuneus (autobiographic memory). Interestingly, both instructions also revealed a non-significant overlap in the posterior medial frontal cortex (pmFC, including the dorsal part of the anterior cingulate cortex—dACC) and the right PFC. These latter two areas are part of a domain-general conflict monitoring network (Botvinick et al., 2004). Botvinick et al. (2004) proposed that this network detects and resolves conflicts between multiple inputs, and suggested that the pmFC detects inconsistencies, while the lateral PFC resolves these by modifying the attention to the different conflicting inputs.

The findings converge on the notion that under explicit processing, implicit information is enriched by retrieving similar behaviors from the past and imaging more vividly social cues on human action (Ma et al., 2011, 2012a), or by taking in more situational information so that a biased attribution in favor of the person is avoided (Kestemont et al., 2013). However, a limitation of current neuroimaging studies on social mentalizing is that they do not present direct evidence to support the interpretation that additional brain activation during explicit judgments reflect verification or correction after a default intuitive response. There is, however, some neuroimaging evidence on racial attitudes demonstrating this correction process, to avoid stereotypic biases. When White egalitarian-motivated people were confronted with Black out-group faces (Cunningham et al., 2004; Forbes et al., 2012), the amygdala was activated only under short (~30 ms) but not under long presentations times (~500 ms). The finding that amygdala activation correlated with a behavioral test of racial attitudes suggests that the initial amygdala response was biased, while later explicit processing reevaluate out-group members in a non-biased manner.

## Implicit and explicit mentalizing as iterative reprocessing

Taken together, recent neuroscientific data on the neural underpinnings of social mentalizing suggest that implicit and explicit mentalizing share the same early timing and the same core brain areas, but also that explicit inferences may lead to a modulation in some brain areas, reflecting a correction or an enrichment. These data are best explained by more recent *default-interventionist* dual-process theories (Evans, 2011). These dual-process theories argue that implicit processes may provide a quick default solution to an assessment (resulting in an identical timing of early inferences), which may afterwards be either accepted or corrected by explicit reasoning (resulting in longer processing times overall). Cunningham and Zelazo (2007) proposed an interesting implementation of this idea which they termed *iterative reprocessing*. According to these authors, processing occurs on a continuum from relatively implicit to relatively explicit. Increased explicit processing is possible through additional reprocessing cycles, which enables more explicit elaboration of information along a wider and richer range of contexts and constraints, retrieving input from increasingly more brain structures. Inferences are hierarchically arranged, so that lower-level processes continuously provide valenced information, while higher-order processes recruited during subsequent cycles render the inference more explicit. With each iteration cycle, information is passed back and forth between the lower-level and higher-level computations (Cunningham et al., 2007). According to some theories of consciousness, these additional cycles bring the inference process closer to a steady neural attractor state that is accessible to introspection (Dennett, 2001; Timmermans et al., 2012). This iterative cycling theory has been implemented in multilevel bidirectional connectionist computer models of person construal (Freeman and Ambady, 2011) and attitude formation (Ehret et al., unpublished), in which a continuous interaction between lower and higher-level social information dynamically leads to stable person construals (Freeman and Ambady, 2011) and attitudes about them (Ehret et al., unpublished).

It is suggested here that social mentalizing involves similar iterative reprocessing from implicit to explicit (Cunningham and Zelazo, 2007; Freeman and Ambady, 2011). Inferences based on one or a few cycles are relatively implicit and crude intuitions, leaving an early mark in the ERPs and activation in restricted core brain areas. In contrast, inferences based on additional iterations and computations are increasingly rich, balanced and relatively explicit, leaving a broader trace of activation in multiple brain areas. Social inferences are hierarchically arranged, with lower-level brain areas (e.g., amygdala) continuously providing valenced information (Todorov et al., 2008a,b; Vandekerckhove and Panksepp, 2011) and moderate-level interpretations of behaviors (e.g., TPJ) sending information on the agent's intentions, which feeds higher-level interpretations of the agent in terms of traits (e.g., mPFC; Van Overwalle, 2009; Ma et al., 2012b). These higher-order inferences are recruited during additional processing cycles which make it more likely that situational information is considered and biases are corrected.

How these subsequent cycles touch on novel input and memories, and how this information is integrated and feed back to implicit intuitions at the neural level, are questions for future research. Moreover, it is likely that individual differences in motivation or need for cognition (Epstein et al., 1996; Roets and Van Hiel, 2007, 2011) determine how much cycles are processed until an inference is terminated. Or does this depend more on situational opportunities?

Apart from these basic questions on iterative reprocessing of implicit and explicit mentalizing (Cunningham and Zelazo, 2007; Freeman and Ambady, 2011), there are also novel neuroscientific findings that can be viewed from this perspective. For instance, recent research demonstrated that the ventral mPFC locates a trait code (Ma et al., 2013), that is, a distributed memory that encodes trait information and, when activated, enable access to this stored information (Wood and Grafman, 2003; Northoff and Bermpohl, 2004). The trait code is assumed to have several links to typical behaviors that exemplify the trait, and so facilitate easy understanding and trait attribution on the basis of behavior alone. This suggests that the trait code allows easy access already during early implicit processing (as an intuition). As another example, novel evidence on the role of the dorsal mPFC reveals that this area is strongly involved in generating high construals, that is, abstract categories extracted from lower-level behavior or object information (Baetens et al., 2013). As such, it appears to be the ideal candidate for high-level abstraction in an iterative reprocessing framework. An obvious prediction is that this abstraction function is mainly accessible in explicit as opposed to implicit processing, because only repetitive cycling might allow to reach complex abstractions and awareness about them. Finally, there is also novel evidence on the role of the dorsal mPFC in social working memory (Meyer et al., 2012). Keeping an increasing number of individuals in mind, also increased activity of the dorsal mPFC. Future research can explore how social working memory fits with an iterative reprocessing framework.

## Conclusion

Previous neuroscientific research has demonstrated that understanding of another persons' mind involves both implicit and explicit processes located in the mentalizing network (e.g., Keysers and Gazzola, 2007). The old idea still present in some dual-process theories that implicit and explicit thought is inflexibly driven by entirely different underlying systems and brain areas (Smith and DeCoster, 2000; Strack and Deutsch, 2004) is contradicted by recent ERP and fMRI findings on mentalizing. Under implicit and explicit processing instructions, there was a shared early timing (ERP studies) and shared brain activity (fMRI studies) pointing to a single core system of social mentalizing (Van Overwalle, 2009). These data can be explained by a default-interventionist dual process (Evans, 2008) in terms of iterative reprocessing (Cunningham and Zelazo, 2007; Cunningham et al., 2007; Freeman and Ambady, 2011). According to this framework, there is an implicit default core process that allows observers to make quick social mentalizing inferences, presumably based on pre-existing learned social knowledge. This implicit core process is subserved by the TPJ and mPFC. Subsequent reprocessing cycles allow to take in more and richer information which enable observers to verify and flexibly correct their original rapid intuition.

### Conflict of interest statement

The authors declare that the research was conducted in the absence of any commercial or financial relationships that could be construed as a potential conflict of interest.
